# Takotsubo Syndrome and Gender Differences: Exploring Pathophysiological Mechanisms and Clinical Differences for a Personalized Approach in Patient Management

**DOI:** 10.3390/jcm13164925

**Published:** 2024-08-21

**Authors:** Simona Giubilato, Giuseppina Maura Francese, Maria Teresa Manes, Roberta Rossini, Roberta Della Bona, Laura Gatto, Antonio Di Monaco, Filippo Zilio, Nicola Gasparetto, Carlotta Sorini Dini, Francesco Borrello, Antonia Mannarini, Angela Beatrice Scardovi, Daniela Pavan, Francesco Amico, Giovanna Geraci, Carmine Riccio, Furio Colivicchi, Massimo Grimaldi, Michele Massimo Gulizia, Fabrizio Oliva

**Affiliations:** 1 Cardiology Department, Cannizzaro Hospital, 95126 Catania, Italy; 2 U.O.C. Cardiologia, Ospedale Garibaldi-Nesima, Azienda di Rilievo Nazionale e Alta Specializzazione “Garibaldi”, 95100 Catania, Italy; 3 Cardiology Division, St Francesco Hospital, 87027 Paola, Italy; mteresa.manes@aspcs.it; 4 SC Cardiologia, Azienda Santa Croce e Carle, 12100 Cuneo, Italy; 5 Cardiovascular Disease Unit, IRCCS Ospedale Policlinico San Martino, 16100 Genoa, Italy; roberta.dellabona@gmail.com; 6 Cardiology Department, San Giovanni Addolorata Hospital, 00184 Rome, Italy; 7 Department of Cardiology, General Regional Hospital “F. Miulli”, Acquaviva delle Fonti, 70021 Bari, Italy; a.dimonaco@gmail.com (A.D.M.); m.grimaldi@miulli.it (M.G.); 8 Department of Cardiology, Santa Chiara Hospital, APSS, 38121 Trento, Italy; filippo.zilio@apss.tn.it; 9 Division of Cardiology, AULSS2 Marca Trevigiana, Ca’ Foncello Hospital, 31100 Treviso, Italy; 10 U.O.C. Cardiologia Clinico Chirurgica (UTIC), A.O.U. Senese Ospedale Santa Maria alle Scotte, 53100 Siena, Italy; carlotta.sorinidini@gmail.com; 11 Division of Cardiology and Intensive Care Unit, Pugliese-Ciaccio Hospital, 88100 Catanzaro, Italy; 12 Division of Cardiology, Cardiothoracic Department, Azienda Consorziale Ospedaliera-Universitaria, 70124 Bari, Italy; 13 U.O.C. Cardiologia, Ospedale S. Spirito, 00123 Roma, Italy; angela.scardovi@aslroma1.it; 14 Cardiology Unit, Azienda Sanitaria “Friuli Occidentale”, 33170 Pordenone, Italy; 15 Cardiology Unit, S. Antonio Abate Hospital, ASP Trapani, 91016 Erice, Italy; 16 Cardiovascular Department, Sant’Anna e San Sebastiano Hospital, 81100 Caserta, Italy; 17 Cardiology Department, San Filippo Neri Hospital, ASL Roma 1, 00135 Rome, Italy; 18 “A. De Gasperis” Cardiovascular Department, Division of Cardiology, ASST Grande Ospedale Metropolitano Niguarda, Piazza dell’Ospedale Maggiore 3, 20162 Milan, Italy

**Keywords:** takotsubo syndrome, broken heart syndrome, gender difference, diagnostic algorithm, management

## Abstract

Takotsubo syndrome (TTS), also known as the broken-heart syndrome, is a reversible condition typically observed in female patients presenting for acute coronary syndromes (ACS). Despite its increasing incidence, TTS often remains undiagnosed due to its overlap with ACS. The pathophysiology of TTS is complex and involves factors such as coronary vasospasm, microcirculatory dysfunction, increased catecholamine levels, and overactivity of the sympathetic nervous system. Diagnosing TTS requires a comprehensive approach, starting with clinical suspicion and progressing to both non-invasive and invasive multimodal tests guided by a specific diagnostic algorithm. Management of TTS should be personalized, considering potential complications, the presence or absence of coronary artery disease (CAD), diagnostic test results, and the patient’s clinical course. The current data primarily derive from case series, retrospective analyses, prospective registries, and expert opinions. In recent years, there has been growing recognition of gender differences in the pathophysiology, presentation, and outcomes of TTS. This review provides an updated overview of gender disparities, highlighting the importance of tailored diagnostic and management strategies.

## 1. Introduction

Takotsubo syndrome (TTS), also called stress-induced cardiomyopathy, is a clinical entity characterized by an acute onset and transient myocardial stunning, leading to temporary regional left ventricular systolic dysfunction [1,2]. Given its transient nature, the most recent ESC guidelines on cardiomyopathies recommend excluding TTS from the classification of cardiomyopathies [3]. The term “Takotsubo” derives from the balloon-like shape, known as “apical ballooning”, which the apex of the left ventricle assumes in the majority of TTS cases. This distinctive shape can be visualized using several imaging modalities including echocardiography, ventriculography, and cardiac magnetic resonance imaging (CMR). The shape resembles a traditional Japanese octopus trap called “tako-tsubo”, hence the name TTS. The syndrome was first described in Japan in the 1990s [4].

A defining characteristic of TTS is the presence of a trigger, observed in around two-thirds of patients, often following emotional or physical stress [5]. Emotional stressors typically involve negative life events such as bereavement, job loss, financial difficulties, interpersonal conflicts, depression, or post-traumatic stress disorder. Physical stressors may include a range of medical conditions affecting various organ systems, such as stroke, intracranial hemorrhage, exacerbations of chronic obstructive pulmonary disease (COPD), pheochromocytoma, gastrointestinal bleeding, neoplasms, or surgical procedures. Additionally, individuals with TTS frequently exhibit chronic anxiety disorders or a familial history of psychiatric conditions [6]. Notably, TTS may also manifest in the absence of an identifiable trigger in approximately one-third of cases.

## 2. Epidemiology and Gender Difference in TTS Incidence and Triggers

Epidemiological data reveal that TTS accounts for approximately 2% of all ACS cases and 0.02% of all hospital admissions in the United States [7]. However, during the COVID-19 pandemic, there was a significant increase in the incidence of TTS [8]. Furthermore, with respect to the true global burden of TTS, it is possible that some patients with TTS go undiagnosed in centers that lack the capacity for differential diagnosis. Interestingly, TTS is nine times more common in women in Western countries than in Japan, where TTS is more commonly diagnosed in men. In Western countries, the incidence rises to approximately 10% when looking specifically at female cohorts, with 80% of these women being post-menopausal and having an average age between 67 and 70 years [7]. In contrast, there is no significant correlation between age and the onset of TTS in males, who are typically diagnosed at a younger age [9,10]. Moreover, TTS is not limited to adult patients and has also been reported in children, including premature neonates [11,12]. TTS is infrequently reported among African American and Hispanic populations, with many documented cases involving individuals of Caucasian descent [7,13].

**Figure 1:**
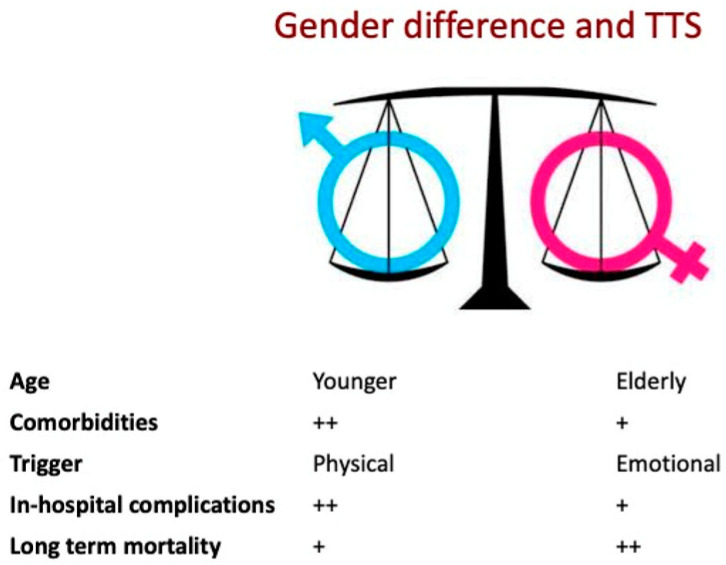
Main gender differences in TTS. “+” sign indicates “present” and the “++” sign indicates “more frequent”.

Additionally, the prognosis of TTS in patients appears to be influenced by ethnicity, with higher rates of in-hospital complications observed among African American and Japanese patients compared to Caucasian and Hispanic patients [14,15]. There is conflicting evidence regarding potential seasonal variations in TTS. Nevertheless, several studies have identified a seasonal variation pattern in TTS that is in contrast to ACS, with peaks during the summer [16,17,18]. The precise mechanisms underlying gender differences in TTS remain unclear. However, the increased incidence of post-menopausal cases suggests that estrogens may have a cardioprotective role against the onset of TTS. Emotional triggers are more commonly associated with TTS in women, whereas men tend to have a higher prevalence of TTS cases triggered by physical stressors, such as acute medical conditions or surgical procedures. Figure 1 illustrates the main gender differences in TTS. Notably, the age at presentation and type of stressor are comparable to other uncommon causes of ACS, including myocardial infarction with non-obstructed coronary arteries and spontaneous coronary artery dissection [19,20,21]. This suggests that hormonal and physiological factors may influence the triggers of TTS in different sexes [22,23]. Further research is needed to fully understand these differences.

## 3. TTS Types

Although several anatomical variants of TTS have been described, four principal types are distinguished based on the regional distribution of segmental kinetics deficits of the left ventricle [9]. The typical pattern, present in approximately 80% of cases, is characterized by apical ballooning with apical hypokinesia, akinesia, dyskinesia, and basal hyperkinesia. The atypical forms of TTS include the mid-ventricular form (4–40% of cases), focal forms typically involving anterolateral segments (1.5–7% of all cases), and basal forms (1–3%). Basal forms are particularly common in patients with pheochromocytoma and intracranial hemorrhaging, conditions that should be investigated when this atypical form of TTS is present. In addition to the four main variants, cases with biventricular or isolated right ventricular involvement, which are found in approximately one-third of cases, have been described and are associated with a worse prognosis in the short- and long-term. These patients are generally younger and have less impairment of left ventricular ejection function (LVEF). However, LVEF usually recovers to normal levels regardless of the initial form of TTS, and different forms of TTS can occur in the same patient. No specific differences between typical and atypical TTS have been described in terms of symptoms on admission and emotional or physical triggering factors. Brain natriuretic peptide (BNP) and C-reactive protein (CRP) levels are higher in patients with typical TTS than in those with atypical TTS. Conversely, troponin levels do not differ between the groups.

Regarding electrocardiographic abnormalities, typical TTS is more often associated with atrial fibrillation, ST-segment elevation, and T-wave inversion. In contrast, ST-segment depression is more common in patients with atypical TTS, especially in those with the basal form, who also typically have significantly longer QTc times.

There is no significant difference in complication rates between typical and atypical TTS during the in-hospital phase, with a similar incidence of cardiogenic shock and death. Long-term follow-up also shows comparable rates of major adverse cardiac and cerebrovascular events in patients with typical and atypical TTS. However, Ghadri et al. demonstrated a slightly increased mortality rate in patients with typical TTS in the first year after which the mortality rates are similar [24]. Nevertheless, multivariate analysis indicates that the type of TTS is not an independent predictor of mortality at 1 year. Instead, factors such as LVEF less than 45%, atrial fibrillation, and the presence of neurological disease are significant predictors [24].

## 4. Pathophysiological Mechanisms

**Figure 2:**
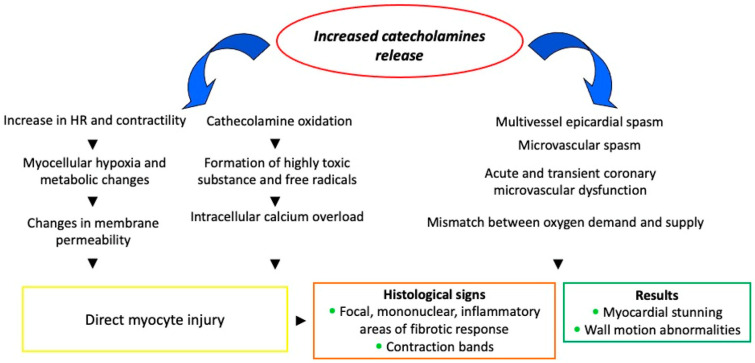
Pathophysiological mechanisms of TTS.

Although the underlying pathophysiological causes of the syndrome are not yet fully understood, there is evidence to suggest that multiple mechanisms may contribute to its development, with no single mechanism being sufficient to explain its development in isolation (Figure 2). The risk factors for TTS include estrogen deficiency, emotional or physical stress, and genetic predisposition.

For a long time, the most widely accepted theory was that of catecholamine-induced cardiotoxicity [25,26]. Several studies have observed critically elevated plasma levels of catecholamines in patients with TTS, with serum concentrations two to three times higher than those in patients with ACS.

Elevated levels of catecholamines, produced by the sympathetic nervous system in response to stress, can lead to intracellular calcium overload and cardiac dysfunction through the β-adrenergic receptor signaling pathway. This calcium overload in myocardial cells could cause ventricular dysfunction and catecholamine-induced cardiotoxicity, similar to that observed in pheochromocytoma. Cardiotoxicity is characterized by significant changes in myocardial characteristics, including contraction band necrosis, inflammatory cells infiltration, and fibrosis [27].

Another widely studied mechanism is microvascular dysfunction [28]. Numerous studies have shown impaired endothelium-dependent vasodilation in TTS patients, with excessive vasoconstriction and reduced myocardial perfusion. It has been hypothesized that myocardial dysfunction is the result of compromised microvascular perfusion, leading to an oxygen demand–supply mismatch and subsequent myocardial ischemic stunning [29]. Consequently, risk factors for endothelial dysfunction could predispose individuals to TTS. It remains unclear whether the compromised myocardial perfusion, documented during episodes of stress in TTS, is the cause or the effect of myocardial stunning.

Recent advances in neuroimaging techniques have suggested that neurohumoral connections between the heart and brain provide the anatomical and functional substrate underlying the clinical onset of TTS. These connections involve the cognitive centers of the brain and the hypothalamic–pituitary–adrenal axis [30,31].

Before TTS was recognized as a separate syndrome, an increased incidence of transient cardiac dysfunction associated with dynamic ECG changes and a greater risk of arrhythmias was observed in patients with acute stroke, especially hemorrhagic strokes involving the basal ganglia or brainstem. This is a transient phenomenon, known as “neurogenic myocardial stunning”. According to more recent theories, stress factors activate the brain and neurohormonal axis, resulting in elevated cortisol levels, increased catecholamine bioavailability, and the release of neuropeptides involved in regulating stress responses, such as neuropeptide Y (NPY), which is synthesized in the arcuate nucleus of the hypothalamus [32,33]. This process initiates myocardial injury through several pathophysiological mechanisms, such as direct toxic effects of catecholamines, adrenoceptor-mediated damage, microvascular and epicardial coronary vasoconstriction and/or spasm, and increased workload. In addition, other risk factors, such as estrogen deprivation, may facilitate TTS, possibly through endothelial dysfunction.

One of the most significant features of TTS is its strong correlation with preceding stressful events. Research indicates that both emotional and physical stressors play a crucial role in triggering TTS. A wide range of emotional and physical triggers have been reported. Physical triggers are more common than emotional triggers, especially in male patients, whereas an emotional trigger is more common in females [34]. Emotional triggers include divorce, burglary, job loss, debt, death of a family member or of partner, arguments, earthquakes, storms, car accidents, or rarely, happy events (“happy heart syndrome”) such as winning the jackpot, a wedding, or the birth of a grandchild [34]. Interestingly, happy heart syndrome is characterized by a higher prevalence in male patients with atypical, non-apical ballooning variant compared to patients with negative emotional stressors [35]. Physical triggers include, for example, cerebral bleeding, stroke, TIA, pneumonia, pulmonary embolism, cancer, sepsis, fracture, surgery, and anesthesia, while pheochromocytoma can cause TTS by the direct production of catecholamines. Lastly, psychiatric disorders such as anxiety, depression, suicide attempts, and post-traumatic stress disorder are associated with TTS [34,36].

However, the understanding of why certain psychological and/or physical stressors lead to the development of TTS in some individuals and not in others is still inadequate.

Furthermore, a stressed heart morphology represented by basal septal hypertrophy due to undiagnosed hypertension associated with chronic emotional stress may predispose to the development of TTS [37,38].

Current research has illustrated a role that predisposing genetic factors have in the onset of TTS, linked to specific gene polymorphisms involved in an intracellular response to beta-adrenergic receptor stimulation [39,40]. Several studies are now underway to investigate the possible genetic associations (GENETIC [Is There a Genetic Pre-disposition for Acute Stress-induced {Takotsubo}Cardiomyopathy], NCT04513054).

## 5. Clinical Presentation

The clinical presentation of TTS closely resembles that of ACS. More than 75% of TTS patients experience chest pain, while about half report dyspnea. Less common symptoms include dizziness and syncope. Early studies suggested that TTS was a self-limiting condition with a better prognosis than ACS. However, recent data suggest similar rates of complications and in-hospital mortality, which can be as high as 5% [5,9].

Risk stratification that uses echocardiographic, electrocardiographic, and hemodynamic parameters is essential for all patients with a confirmed diagnosis of TTS. Common complications during the acute phase include acute heart failure (12–45%), cardiogenic shock (6–20%), dynamic obstruction of the left ventricular outflow tract (LVOTO) (10–25%), mitral regurgitation (14–25%), atrial fibrillation (5–15%), major ventricular arrhythmias (ventricular fibrillation and torsades de pointes) (2–8%), advanced atrioventricular blocks (5%), intraventricular thrombosis (especially in apical forms, with possible systemic embolism) (2–8%), interventricular septal rupture, and free wall rupture (<1%) [41].

Several parameters have been identified as predictors of adverse outcomes during the in-hospital phase. These include age over 75 years, male sex, elevated initial levels of troponin and BNP, and the extent of ST-segment elevation.

Major complications, such as arrhythmic complications, apical thrombosis, and ventricular rupture are more frequent in males [9,22,23,42]. This may be related to the higher prevalence of cardiovascular risk factors in men, which may also explain the higher rates of serious adverse cardiac and cerebrovascular events during the in-hospital phase and up to 60 days after the acute event. These findings support closer monitoring during both the in-hospital phase and follow-up period, particularly among male patients.

Additionally, several echocardiographic indices, including LVEF < 45%, elevated E/e’ ratio, moderate/severe mitral regurgitation, and right ventricular involvement, are considered predictors of adverse outcomes [43,44].

There are few studies on the long-term outcomes of TTS patients. However, the available literature suggests that annual morbidity and mortality rates are similar to those of patients with coronary artery disease (CAD), challenging the notion of a favorable long-term prognosis for TTS [45,46].

An important determinant of long-term prognosis seems to be the underlying trigger factor for TTS onset. In a recent study, Ghadri et al. proposed a new classification of TTS (InterTAK Classification) based on the precipitating factor (Table 1) [47]. The authors observed that patients whose TTS was triggered by neurological pathologies experienced higher complication rates and poorer prognoses compared to those whose TTS was precipitated by physical stress, medical conditions, or procedures. Patients with TTS triggered by emotional stress and those with no identifiable trigger events had more favorable outcomes.

## 6. Diagnosis

### 6.1. Diagnostic Criteria

The diagnosis of TTS is particularly challenging because its clinical, instrumental, and laboratory presentations can resemble those of ACS according to symptoms, electrocardiographic changes, and the increase in myocardial necrosis markers. Nonetheless, specific diagnostic criteria have been developed to differentiate TTS from ACS, myocardial infarction with non-obstructive coronary arteries (MINOCA), and myocarditis.

Abe et al. first proposed the initial diagnostic criteria for TTS in 2003 [48]. Subsequently, in 2008, researchers at the Mayo Clinic refined these criteria, which include transient hypokinesia, akinesia, or dyskinesia of the mid-segments of the left ventricle, with or without involvement of the apex [49]. It is important to emphasize that segmental kinetic abnormalities must extend beyond the distribution of a single epicardial coronary artery and are often associated with a stressful event as a trigger, although this circumstance may not always be present. Additional criteria include non-obstructive CAD or angiographic evidence of coronary plaque rupture, newly observed electrocardiographic abnormalities (ST-segment elevation and/or T-wave inversion), a slight rise in markers of myocardial necrosis, and the absence of pheochromocytoma and myocarditis.

In 2017, data from the InterTAK international registry were used to create the InterTAK Diagnostic Score [50], which supports the differential diagnosis between TTS and ACS in patients who are admitted to the emergency room with symptoms. This score considers five historical variables (female sex, emotional stress, physical stress, neurological disorders, and psychiatric disorders) and two electrocardiographic variables (absence of ST-segment depression and prolongation of QTc interval). A score greater than 50 indicates a high probability of TTS, while a score less than 31 suggests a high probability of ACS (Table 2).

The latest International Takotsubo Diagnostic Criteria (InterTAK criteria) were presented in 2018 as an international consensus document and include the following [34]:

1. Transient left ventricular dysfunction: This can manifest as hypokinesia, akinesia, or dyskinesia with classic apical ballooning or abnormalities involving mid and/or basal segments or focal types. Involvement of the right ventricle may also occur. Transition forms between different patterns are possible, with segmental kinetics abnormalities typically extending beyond a single epicardial vessel. In rare cases, myocardial dysfunction may correspond to the territory supplied by a single coronary artery (focal TTS). These abnormalities may persist for a prolonged period, and in some cases, recovery of contractility may never be documented.
2. Stress factors: Emotional, physical, or combined stress may precede the onset of TTS, but the absence of a stressful event does not rule out the diagnosis.
3. Neurological disorders: Subarachnoid hemorrhage, stroke/TIA, or seizures and pheochromocytoma can trigger TTS.
4. ECG: New changes such as ST-segment abnormalities, T-wave inversion, and QTc interval prolongation are common, though TTS can occur without these alterations.
5. Cardiac biomarkers: Troponin and creatine kinase levels are moderately elevated in the majority of cases, while a notable rise in BNP or NT-proBNP levels is common.
6. CAD: The presence of obstructive CAD identified through coronary angiography does not rule out TTS.
7. Exclusion of myocarditis: The existence of myocarditis must be excluded with CMR which is recommended for the differential diagnosis between myocarditis and TTS.
8. Demographics: TTS predominantly affects post-menopausal women.

The key updates to the previous criteria include recognizing focal TTS as a morphological variant and the addition of pheochromocytoma as a potential trigger.

Significant CAD coexists in 10–30% of all TTS cases and is considered an insufficient criterion to exclude the TTS diagnosis [51,52]. The relationship between segmental kinetics abnormalities and territories of epicardial vessel distribution must be considered in the differential diagnosis.

**Figure 3:**
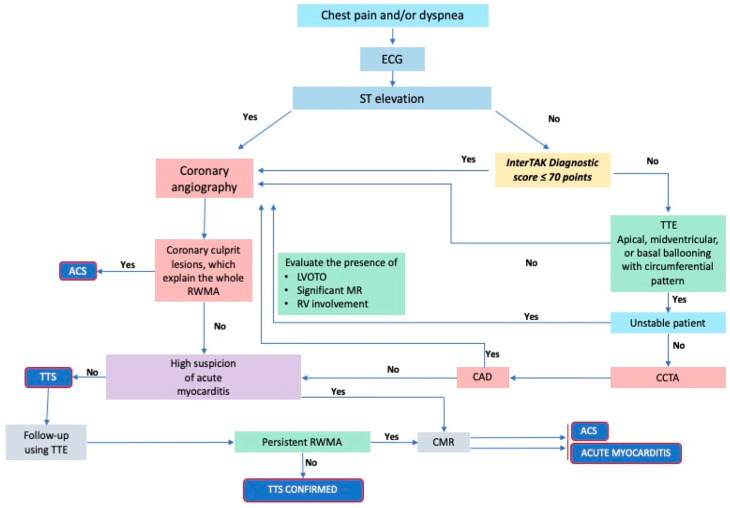
Diagnostic algorithm for TTS, adapted from Ghadri, J.R., et al. *Eur. Heart J.* 2018 [53].

The same authors developed a diagnostic algorithm that incorporates various clinical and instrumental parameters, including the InterTAK score [53], to differentiate between TTS, ACS, and myocarditis (Figure 3). The first diagnostic step is to assess the presence or absence of ST-segment elevation on the ECG. Patients with ST-segment elevation should undergo coronary angiography to exclude myocardial infarction. A coronary angiography is also indicated for patients without ST-segment elevation, with a low likelihood of TTS based on the InterTAK score or for unstable patients, in whom a transthoracic echocardiogram, is critical to identify potential complications associated with TTS, including the occurrence of LVOTO, development of severe mitral regurgitation, or wall rupture. In stable patients or those with a high probability of TTS, the diagnostic workup includes a transthoracic echocardiogram and coronary computed tomography angiography (CCTA), if promptly available.

This algorithm indicates that TTS is the most likely diagnosis for patients with typical echocardiographic pattern, normal coronary arteries on coronary angiography or CCTA, a negative medical history, and clinical-instrumental parameters that rule out myocarditis. If the differential diagnosis with ACS and myocarditis remains uncertain, CMR with late gadolinium enhancement (LGE) should be included in the diagnostic workup. Follow-up monitoring is essential for evaluating the potential functional recovery of segmental kinetic abnormalities and left ventricular function, as well as for confirming the diagnosis of TTS. This can be achieved through imaging modalities, such as transthoracic echocardiography and/or CMR [53,54].

Acronyms in Figure 3: ACS: acute coronary syndromes; CAD: coronary artery disease; CCTA: coronary computed tomography angiography; CMR: cardiac magnetic resonance; ECG: electrocardiogram; LVOTO: left ventricle outflow tract obstruction, MR: mitral regurgitation; RV: right ventricle; RWMA: regional wall motion abnormality; TTE: trans-thoracic echocardiogram; TTS: Takotsubo syndrome.

### 6.2. Diagnostic Exams

#### 6.2.1. Biomarkers

Patients with TTS present elevated troponin I and T values at onset, similarly to patients with ACS. However, in most cases, the peak troponin values in TTS are lower than those found in ACS [9]. There is generally a discrepancy between the slight increase in myocardial necrosis markers and the extent of cardiac segmental kinetic abnormalities. This discrepancy arises because much of the left ventricular systolic dysfunction is attributable to transient, reversible ventricular stunning. Typically, a slight increase in creatine kinase levels is also observed.

Atrial natriuretic peptides (BNP and NT-proBNP) levels are typically higher in TTS patients compared to those with ACS. Peak values are generally reached 24–48 h after the onset of symptoms and may take several months for these values to fall back within the normal range [55]. The extent of the increase in BNP and NT-proBNP, in contrast to troponin, correlates with the severity of cardiac segmental kinetic abnormalities, the degree of left ventricular dysfunction, and the existence of edema on CMR.

Inflammation may also play a significant role in TTS, as evidenced by elevated CRP levels in TTS patients, which correlate with reduced LVEF and prolonged hospitalization [56].

#### 6.2.2. Electrocardiogram (ECG)

Electrocardiographic abnormalities are detected in more than 95% of patients with TTS. These abnormalities can include several findings such as long QT interval, ST-segment elevation, T-wave inversion, Q waves on the anterior leads due to electrical stunning, low QRS voltages linked to edema; and less frequently, ST-segment depression, left bundle branch block, or atrioventricular blocks.

T-wave inversion, often diffuse and profound, and significant QT interval prolongation, which usually develops 24 to 48 h after symptom onset, are quite specific to TTS and are included in the InterTAK Diagnostic Score [50].

QT interval prolongation can progress over time to exceed 500 ms, predisposing patients to torsade de pointes and ventricular tachycardia/fibrillation (VT/VF).

Ventricular arrhythmias (VA) on admission are present in 15% of TTS patients and are linked with a 22-fold increase in subacute VAs occurrence during in-hospital stays [57]. It is notable that VAs on admission are primarily linked to catecholamine excess-induced calcium overload, which can lead to delayed after-depolarization or an abnormal automaticity. Conversely, the majority of VAs occurring in the subacute phase are predominantly associated with an increased dispersion of repolarization and susceptibility to torsade de pointes.

Recent data clearly indicate an association between subacute VAs and worse prognosis [58] as well as with an increased mortality rate, even during the in-hospital phase [59]. Furthermore, in patients with TTS subacute VAs are associated with repolarization alterations that can be identified on conventional ECG using the T peak–T end interval. The corrected global (mean of the 12-lead ECG values) T peak–T end interval at 48 h from admission is an independent predictor of subacute VAs and outperforms the standard corrected QT interval in prognostic value. A cut-off of 108 msec for the corrected global T peak–T end yields a sensitivity of 71% and specificity of 72% in predicting subacute VAs [59].

Patients with TTS require ECG telemetry monitoring for at least 48–72 h or until QT interval prolongation resolves [60].

Approximately 40% of TTS cases involve ST-segment elevation in the precordial leads. This elevation typically involves leads V2 to V5, aVR and DII, whereas in anterior STEMI, it usually involves leads V1 to V4, DI, and AVL. It is very rare for the ST-segment elevation to be limited to the inferior leads. Various ECG criteria have been proposed to discriminate with higher sensitivity and specificity between TTS and anterior STEMI. However, in case of ST-segment elevation, it is mandatory to proceed with an urgent coronary angiographic study to differentiate myocardial infarction from TTS.

ST-segment depression is rare in TTS, occurring in less than 10% of TTS patients, compared to over 30% in ACS patients, which makes its presence more suggestive of ACS.

Like ACS, ECG abnormalities in TTS are dynamic. They resolve over time, with a progressive resolution of the ST-segment elevation and T-wave inversion, and a gradual normalization of QT interval over time.

#### 6.2.3. Echocardiography

Echocardiography is the most useful imaging tool for evaluating abnormalities in the segmental kinetics of the left ventricle in TTS. It allows for the identification of different variants of TTS (apical, mid-ventricular, basal, and focal), the assessment of right ventricle involvement, and the detection of potential complications in unstable patients. The echocardiographic recognition of the classic “apical ballooning” variant, which is highly suggestive of TTS, is often associated with other echocardiographic signs of TTS. These include LVOTO due to hypercontractility of basal segments, systolic anterior motion (SAM) of mitral leaflets, and consequent functional mitral regurgitation, which can be assessed using continuous Doppler. The use of contrast-enhanced echocardiography may facilitate the diagnosis of TTS, particularly for a more accurate evaluation of the LV apex, especially in patients where the window for imaging is suboptimal. In TTS, the segmental kinetic abnormalities of the left ventricle extend beyond the distribution of a single coronary artery territory, resulting in a “circular” systolic dysfunction on speckle-tracking echocardiography, with paradoxical longitudinal strain of the mid-apical segments [61]. Recurring echocardiographic evaluations should be performed in a systematic manner in patients with TTS in order to evaluate the occurrence of complications and document the potential recovery of segmental kinetic abnormalities, which typically occur within 4–8 weeks [10].

Additionally, the presence of basal septal hypertrophy on echocardiography, an imaging biomarker for left ventricle remodeling in response to different stressor stimuli, could identify patients at high risk for recurrence [37,38].

#### 6.2.4. Coronary Angiography and Ventriculography

Coronary angiography and ventriculography are commonly performed in most patients with suspected TTS to exclude the development of ACS. Nevertheless, an invasive evaluation is not necessary for all patients with suspected TTS. The decision to proceed with diagnostic coronary angiography should be made on a case-by-case basis, considering a number of factors such as the presence or absence of significant ST-segment abnormalities on ECG, the pre-test probability of TTS according to the Inter-TAK score, and the clinical presentation. This is in accordance with the recommendations set out in the diagnostic algorithm outlined in the latest consensus document on TTS [42]. The absence of significant stenosis or coronary occlusions permits the exclusion of ACS and the confirmation of the diagnosis of TTS. It is worth mentioning that a variable percentage of TTS patients, ranging from 10% to 29%, may have coexisting significant CAD. This does not automatically rule out the diagnosis of TTS. Furthermore, TTS can coexist with myocardial infarction, and myocardial infarction itself can also act as a trigger for the onset of TTS [40,41].

In the differential diagnosis, it should be considered that, in contrast to myocardial infarction, which typically affects a specific territory of the myocardium based on the involved coronary artery, TTS usually involves regional myocardial kinetic abnormalities extending beyond the distribution of a single coronary artery. In most cases of where TTS is suspected, ventriculography should be performed, unless there are contraindications (e.g., suspicion of apical thrombus) to document the characteristic conformation of the left ventricle and confirm the diagnostic suspicion. This is particularly important in the mid-ventricular form, which may pose more difficulty in visualization on echocardiography.

In the OCTOPUS study (Optimized Characterization of Takotsubo Syndrome by Getting Pressure-Volume Loops), a conductance catheter was inserted into the left ventricle to demonstrate that TTS is characterized by severely compromised cardiac contractility and a shortened systolic period [62]. In response, the heart compensates by increasing left ventricular end-diastolic volume to preserve cardiac output. Diastolic function is marked by prolonged active relaxation but unchanged passive elastic properties. These hemodynamic changes provide insights into the underlying mechanisms of TTS and suggest potential treatment strategies.

#### 6.2.5. Coronary Computed Tomography Angiography (CCTA)

Coronary computed tomography angiography (CCTA) is a non-invasive method for ruling out culprit coronary lesions in stable patients without ST-segment elevation. This approach is especially useful in cases where echocardiographic and electrocardiographic abnormalities, along with elevated markers of myocardial necrosis and atrial natriuretic peptides, occur concurrently with physical triggers commonly associated with the onset of TTS (sepsis, surgical procedures, stroke, subarachnoid hemorrhage, etc.) [53].

#### 6.2.6. Cardiac Magnetic Resonance Imaging (CMR)

Cardiac magnetic resonance imaging (CMR) is a valuable diagnostic tool for identifying TTS as well as differentiating it from other cardiac conditions like ACS and myocarditis. This imaging technique offers comprehensive insights that assist in the differential diagnosis, including the identification of segmental kinetic abnormalities, left and right ventricular systolic function, and myocardial tissue characterization. In order to diagnose acute TTS using CMR, it is necessary to document the presence of segmental kinetic abnormalities, tissue edema, and the absence of LGE [63,64,65,66].

The acute phase of TTS is characterized in the T2-weighted sequences by myocardial edema, manifesting as a high signal intensity in areas exhibiting regional ventricular wall motion abnormalities. In contrast, edema in ACS follows the distribution territory of epicardial coronary arteries, while in myocarditis, it tends to have a non-ischemic distribution, often localized at the basal and lateral segments with subepicardial involvement. In general, the inability to detect macroscopic fibrosis, indicated by the absence of LGE, is a distinctive feature of TTS. This helps to differentiate it from ACS, where LGE is consistently present with varying degrees of subendocardial or transmural involvement, and from myocarditis, where 88% of patients exhibit a “patchy” distribution of LGE. The complete absence of LGE is a highly predictive indicator of complete recovery of kinetic and systolic function abnormalities. However, evidence of LGE in the acute phase of TTS, although typically of lesser intensity compared to ACS, has been documented and is associated with a less favorable outcome [65]. Regarding the timing, it is noteworthy that performing CMR within 7 days of admission for MINOCA appears to yield a higher diagnostic rate [67]. This is consistent with data indicating a higher incidence of normal CMR results in patients who undergo the scan 14 days or more after presentation, likely due to the resolution of edema [68]. A recent study by Shanmuganathan et al. demonstrated that even an early CMR strategy (median 33 h post-admission and 4 h before coronary angiography) can effectively identify the cause of a suspected ACS, including TTS, which accounted for 3% of cases [69]. Therefore, CMR should be performed early in the diagnostic workup, ideally within 2 weeks of presentation.

## 7. Management and Treatment

To date, there are no established guidelines for the treatment of TTS patients. Furthermore, there are currently no prospective randomized trials that have assessed the effect of specific drugs on the prognosis of TTS. Consequently, therapeutic strategies are based on clinical experiences documented in the literature and consensus documents.

Given the high incidence of complications that may arise during the acute phase, approximately 20% of TTS patients, and the potential of these complications to result in electrical or hemodynamic instability, it is recommended that patients with TTS undergo continuous multiparametric monitoring in cardiac intensive care units for a period of 48–72 h, regardless of the presence of ST-segment elevation. During this monitoring phase, risk stratification should be performed based on medical history, clinical parameters, laboratory findings, and instrumental test results. Additionally, it has been observed that complications during the acute phase are more prevalent among male patients, underscoring the importance of providing gender-specific attention and care.

**Figure 4:**
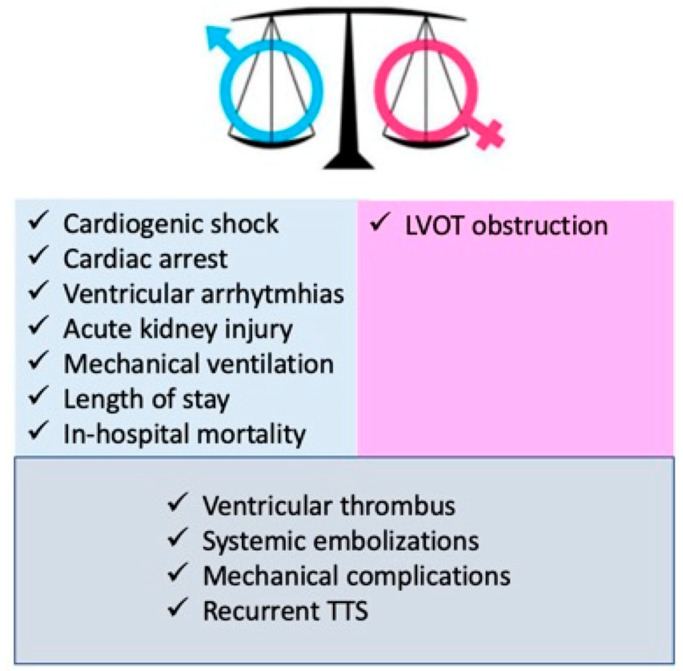
Gender difference in TTS complications.

In particular, men are more likely to develop cardiogenic shock, ventricular arrhythmias, cardiac arrest, and acute kidney injury. Moreover, they are also more likely to require mechanical ventilation, resulting in longer hospital stays and higher in-hospital mortality. Women have a higher incidence of LVOTO due to the smaller left ventricle cavity. Other complications, such as ventricular thrombus, systemic embolization, and recurrent TTS, have a similar distribution between the two sexes [22,23,70,71,72,73]. (Figure 4).

### 7.1. Treatment of Uncomplicated TTS

Beta-blockers and angiotensin-converting enzyme inhibitors (ACEi) or angiotensin II receptor blockers (ARB) are the drugs of choice for patients with uncomplicated TTS. Given the potential role of catecholamine toxicity, it is reasonable to consider the use of beta-blockers until such time as complete recovery of segmental kinetic abnormalities has been achieved. However, studies so far have not demonstrated that beta-blockers improve the prognosis of TTS patients [9,74]. Additionally, due to the possibility of torsades de pointes and atrioventricular block, beta-blockers must be administered with caution in patients exhibiting bradycardia and QTc interval exceeding 500 ms. ACEi and ARB have been demonstrated to facilitate left ventricular recovery, and have been linked to improved post-discharge outcomes. The recurrence rate of TTS is approximately 1–2% per year. A comprehensive meta-analysis has demonstrated that the use of ACEi and ARB instead of beta-blockers can effectively mitigate the probability of recurrences [75]. Accordingly, the current recommendation is that treatment with ACEi/ARB should be continued for a minimum of three months or until complete recovery of segmental kinetic abnormalities has been achieved.

Although the majority of patients with TTS are treated with dual antiplatelet therapy during the acute phase suspected of ACS, current evidence indicates that aspirin and statins should only be administered in patients who are already affected by concomitant CAD or atherosclerosis in other vascular districts (bystander disease). A recent large-scale study in TTS patients did not show any correlation between aspirin use and a reduced risk of major adverse cardiovascular and cerebrovascular events at either 30 days or 5 years [76].

The role of hormone replacement therapy in post-menopausal women, who represent the majority of TTS patients, is still controversial [77]. Psychological or psychotherapeutic support to improve stress management may be particularly beneficial for patients with neuropsychiatric disorders who are at an increased risk of developing TTS.

At present, there is no evidence to suggest that commonly used pharmacological therapies for TTS differ in efficacy based on gender.

### 7.2. TTS Complicated by Left Ventricular Failure

Diuretics are the recommended pharmacological intervention for patients presenting with congestion and no signs or symptoms of cardiogenic shock, in association with beta-blockers and ACEi or ARB. In cases where congestion is accompanied by symptoms but without hypotension, nitrates can be beneficial in reducing left and right ventricular filling pressures and afterload. Non-invasive mechanical ventilation may be necessary for patients with more severe symptoms. In addition, cases requiring mechanical ventilation (invasive positive pressure ventilation or non-invasive positive pressure ventilation) were significantly higher in males [23].

### 7.3. TTS Complicated by Thromboembolism

One of the most severe complications of TTS is thromboembolism, which occurs in 2–14% of TTS patients. The most commonly reported sites of cardio-embolic complications are the renal, cerebral, and peripheral limb arteries [78]. Approximately one-third of patients with left ventricular thrombus (LVT) in TTS experience embolic complications. However, cardio-embolic events can occur even in the absence of detectable LVT [79].

Cases of right ventricular thrombus in patients with biventricular TTS, with or without pulmonary embolism, have also been documented [80].

Patients with severe left ventricular systolic dysfunction and extensive areas of apical akinesia/dyskinesia are at an increased risk of developing intraventricular thrombosis and subsequent systemic thromboembolism.

Management of these patients includes the use of appropriate parenteral antithrombotic prophylaxis (e.g., heparin) to prevent intraventricular thrombus formation, especially during the acute phase when the risk is greatest [81]. If thrombotic formation is detected, oral anticoagulant therapy with warfarin or direct oral anticoagulants must be initiated and continued after discharge until resolution of the left ventricular thrombosis, which is expected to occur along with the resolution of the wall motion abnormalities in approximately 3 months. A large international registry reported a relatively low prevalence of left ventricular thrombi (2.2%), with all the cases presenting with an apical ballooning pattern [82]. A follow-up echocardiography or CMR should be performed to assess thrombus resolution and the recovery of left ventricular function.

### 7.4. TTS Complicated by LVOTO

LVOTO is a possible complication of TTS that occurs in a range from 7% to 25% of cases. It is due to compensatory hypercontractility of the basal segments of the left ventricle, mainly in small ventricles with an asymmetric septal hypertrophy. It is more common in females, due to a small ventricular cavity [22,70,71,72,73].

In patients with TTS who develop the typical form of apical ballooning complicated by LVOTO without cardiogenic shock, the administration of fluids and the use of low-dose, short-acting beta-blockers (e.g., esmolol, metoprolol) may be a beneficial course of action to reduce basal hypercontractility and consequently the degree of obstruction. Nevertheless, the use of beta-blockers is not advised in cases of bradycardia or when LVOTO results in severe left ventricular dysfunction accompanied by hypotension. The administration of ivabradine has been observed to confer certain advantages to patients affected by LVOTO and sinus tachycardia [83]. Conversely, the use of drugs that act on preload and afterload, such as diuretics and nitrates, should be avoided as they have the potential to exacerbate the severity of obstruction. Similarly, the use of an intra-aortic balloon pump, which reduces afterload, is advisable in this context [84,85]. Inotropes, which increase the contractility of the basal segments of the left ventricle, are contraindicated as they may potentially exacerbate the obstruction.

### 7.5. TTS Complicated by Cardiogenic Shock

Cardiogenic shock in TTS is a complication that occurs in about 10% of patients, more frequently in males, as suggested by international registries (19% in males vs. 8% in females in the GEIST registry), and it is associated with a high in-hospital mortality rate [22]. It is recommended that patients with TTS who develop cardiogenic shock should undergo continuous hemodynamic monitoring. It is fundamentally important to ascertain the underlying cause of shock (whether severe left ventricular systolic dysfunction or the presence of LVOTO, etc.) in order to determine the most effective therapeutic options. Generally, the administration of inotropes should be avoided since the additional stimulation of catecholamine receptors may exacerbate a patient’s clinical condition and prognosis, as demonstrated in various studies [8,86]. Although the evidence is limited, levosimendan has shown efficacy and safety in the management of TTS complicated by cardiogenic shock [87,88]. In more severe cases, if medical therapy proves inadequate, it is advisable to consider the use of a mechanical circulatory support device (IMPELLA, ECMO V-A) at the earliest opportunity [89,90].

### 7.6. TTS Complicated by Serious Arrhythmias

In patients affected by TTS, serious arrhythmias, such as VT/VF and complete atrioventricular block, are not uncommon. It is therefore recommended that patients with TTS be monitored by ECG telemetry for a minimum of 48–72 h. Those with serious arrhythmias should be monitored closely throughout their hospital stay and during long-term follow-up. The current literature offers only limited evidence-based guidance on the use of antiarrhythmic drugs in patients with TTS [91]. It is generally recommended that QT-prolonging medications be avoided. The indication for a permanent pacemaker and/or implantable cardioverter–defibrillator in patients with TTS remains a topic of debate, given the reversible nature of cardiac dysfunction in this condition. Recent studies suggest that serious arrhythmias can often be managed with a temporary approach, such as temporary pacing and wearable cardioverter–defibrillators, until recovery of repolarization time and cardiac systolic function [92,93,94]. However, a comprehensive decision-making process is required on a case-by-case basis, taking into consideration the characteristics of the patient, the type of arrhythmic event, and the recovery of systolic function and QT prolongation. Men are more likely to experience VT/VF. This increased incidence of arrhythmias may be due to the higher levels of catecholamines and the greater susceptibility to electrical disturbances in male patients [22,70,71,72,73,95].

## 8. Conclusions

Takotsubo syndrome is a complex disease with diverse pathogenetic mechanisms, clinical presentations, and outcomes, exhibiting significant gender differences. A differential approach is typically employed for diagnosis starting with clinical suspicion and progressing to non-invasive and invasive multimodal tests according to a defined diagnostic algorithm. These tests are crucial for confirming the diagnosis, detecting potential TTS complications, such as LVOTO and cardiogenic shock, and guiding therapeutic decisions. The management of TTS should be tailored to the individual patient. For uncomplicated cases, beta-blockers and ACE inhibitors or ARBs are commonly prescribed. In more severe cases, the administration of vasopressors, inotropes, and mechanical support may be necessary. Additionally, ASA and lipid-lowering agents are recommended for patients with coexisting atherosclerotic disease. Long-term care involves the identification of triggers, regular clinical and imaging follow-up, monitoring for recurrences, and the implementation of cardiac rehabilitation. This review underscores the necessity of acknowledging gender-based differences in the prevalence, clinical manifestation, and outcomes of TTS. Nevertheless, further research is necessary to elucidate the specific factors contributing to these differences and their implications for the development of gender-specific treatment and management strategies for TTS.

## Figures and Tables

**Table 1:** Classification of TTS patients according to causal factors (InterTAK Classification) [47].

	Trigger
Class 1	Emotional stress
Class 2a	Physical stress secondary to physical activities, medical conditions, or procedures
Class 2b	Physical stress secondary to neurological disorder
Class 3	No identifiable triggering factor

**Table 2:** InterTAK Diagnostic Score [50].

	Points
Female sex	25
Emotional trigger	24
Physical trigger	13
Absence of ST-segment depression	12
Prolonged QTc time	6
Psychiatric disorder	11
Neurological disorder	9

## References

[1] Hurst R.T., Prasad A., Askew J.W., Sengupta P.P., Tajik A.J. (2010). Takotsubo cardiomyopathy: A unique cardiomyopathy with variable ventricular morphology. JACC Cardiovasc. Imaging.

[2] Medeiros K., O’connor M.J., Baicu C.F., Fitzgibbons T.P., Shaw P., Tighe D.A., Zile M.R., Aurigemma G.P. (2014). Systolic and diastolic mechanics in stress cardiomyopathy. Circulation.

[3] Arbelo E., Protonotarios A., Gimeno J.R., Arbustini E., Barriales-Villa R., Basso C., Bezzina C.R., Biagini E., Blom N.A., de Boer R.A. (2023). 2023 ESC Guidelines for the management of cardiomyopathies. Eur. Heart J..

[4] Akashi Y.J., Ishihara M. (2016). Takotsubo syndrome: Insights from Japan. Heart Fail. Clin..

[5] Sharkey S.W., Windenburg D.C., Lesser J.R., Maron M.S., Hauser R.G., Lesser J.N., Haas T.S., Hodges J.S., Maron B.J. (2010). Natural history and expansive clinical profile of stress (tako-tsubo) cardiomyopathy. J. Am. Coll. Cardiol..

[6] Summers M.R., Lennon R.J., Prasad A. (2010). Pre-morbid psychiatric and cardiovascular diseases in apical ballooning syndrome (tako-tsubo/stress-induced cardiomyopathy): Potential pre-disposing factors?. J. Am. Coll. Cardiol..

[7] Deshmukh A., Kumar G., Pant S., Rihal C., Murugiah K., Mehta J.L. (2012). Prevalence of Takotsubo cardiomyopathy in the United States. Am. Heart J..

[8] Jabri A., Kalra A., Kumar A., Alameh A., Adroja S., Bashir H., Nowacki A.S., Shah R., Khubber S., Kanaa’n A. (2020). Incidence of Stress Cardiomyopathy During the Coronavirus Disease 2019 Pandemic. JAMA Netw. Open.

[9] Templin C., Ghadri J.R., Diekmann J., Napp L.C., Bataiosu D.R., Jaguszewski M., Cammann V.L., Sarcon A., Geyer V., Neumann C.A. (2015). Clinical features and outcomes of takotsubo (stress) cardiomyopathy. N. Engl. J. Med..

[10] Schneider B., Athanasiadis A., Stöllberger C., Pistner W., Schwab J., Gottwald U., Schoeller R., Gerecke B., Hoffmann E., Wegner C. (2013). Gender differences in the manifestation of tako-tsubo cardiomyopathy. Int. J. Cardiol..

[11] Berton E., Vitali-Serdoz L., Vallon P., Maschio M., Gortani G., Benettoni A. (2012). Young girl with apical ballooning heart syndrome. Int. J. Cardiol..

[12] Otillio J.K., Harris J.K., Tuuri R. (2014). A 6-year-old girl with undiagnosed hemophagocytic lymphohistiocytosis and takotsubo cardiomyopathy: A case report and review of the literature. Pediatr. Emerg. Care.

[13] Nascimento F.O., Larrauri-Reyes M.C., Santana O., Pérez-Caminero M., Lamas G.A. (2013). Comparison of stress cardiomyopathy in Hispanic and non-Hispanic patients. Rev. Espanola Cardiol. (Engl. Ed.).

[14] Imori Y., Kato K., Cammann V.L., Szawan K.A., Wischnewsky M., Dreiding S., Würdinger M., Schönberger M., Petkova V., Niederseer D. (2022). Ethnic comparison in takotsubo syndrome: Novel insights from the International Takotsubo Registry. Clin. Res. Cardiol..

[15] Franco E., Dias A., Koshkelashvili N., Pressman G.S., Hebert K., Figueredo V.M. (2016). Distinctive Electrocardiographic Features in African Americans Diagnosed with Takotsubo Cardiomyopathy. Ann. Noninvasive Electrocardiol..

[16] Manfredini R., Citro R., Previtali M., Armentano C., Patella M.M., Vriz O., Provenza G., Salmi R., Gallerani M., Astarita C. (2009). Summer preference in the occurrence of Takotsubo cardiomyopathy is independent of age. J. Am. Geriatr. Soc..

[17] Song B.G., Oh J.H., Kim H.-J., Kim S.H., Chung S.M., Lee M., Kang G.H., Park Y.H., Chun W.J. (2013). Chronobiological variation in the occurrence of Tako-tsubo cardiomyopathy: Experiences of two tertiary cardiovascular centers. Heart Lung.

[18] Looi J.-L., Lee M., Grey C., Webster M., To A., Kerr A.J. (2018). Seasonal variation in Takotsubo syndrome compared with myocardial infarction: ANZACS-QI 16. N. Z. Med. J..

[19] Ciliberti G., Verdoia M., Musella F., Ceriello L., Scicchitano P., Fortuni F., Zilio F. (2023). MINOCA in Men and Women: Different Conditions and a Single Destiny?. Int. J. Cardiol..

[20] Di Fusco S.A., Rossini R., Zilio F., Pollarolo L., di Uccio F.S., Iorio A., Lucà F., Gulizia M.M., Gabrielli D., Colivicchi F. (2022). Spontaneous coronary artery dissection: Overview of pathophysiology. Trends Cardiovasc. Med..

[21] Zilio F., Muraglia S., Morat F., Borghesi M., Todaro D., Menotti A., Dallago M., Braito G., Bonmassari R. (2021). Sex differences in clinical and angiographic characteristics in spontaneous coronary artery dissection. Futur. Cardiol..

[22] Arcari L., Nunez-Gil I.J., Stiermaier T., El-Battrawy I., Guerra F., Novo G., Musumeci B., Cacciotti L., Mariano E., Caldarola P. (2022). Gender differences in takotsubo syndrome. J. Am. Coll. Cardiol..

[23] Murakami T., Komiyama T., Kobayashi H., Ikari Y. (2022). Gender Differences in Takotsubo Syndrome. Biology.

[24] Ghadri J.R., Cammann V.L., Napp L.C., Jurisic S., Diekmann J., Bataiosu D.R., Seifert B., Jaguszewski M., Sarcon A., Neumann C.A. (2016). Differences in the clinical profile and outcomes of typical and atypical takotsubo syndrome: Data from the International Takotsubo Registry. JAMA Cardiol..

[25] Wittstein I.S., Thiemann D.R., Lima J.A., Baughman K.L., Schulman S.P., Gerstenblith G., Wu K.C., Rade J.J., Bivalacqua T.J., Champion H.C. (2005). Neurohormonal features of myocardial stunning due to sudden emotional stress. N. Engl. J. Med..

[26] Abraham J., Mudd J.O., Kapur N., Klein K., Champion H.C., Wittstein I.S. (2009). Stress cardiomyopathy after intravenous administration of catecholamines and beta-receptor agonists. J. Am. Coll. Cardiol..

[27] Sachdeva J., Dai W., Kloner R.A. (2014). Functional and histological assessment of an experimental model of Takotsubo’s cardiomyopathy. J. Am. Heart Assoc..

[28] Galiuto L., De Caterina A.R., Porfidia A., Paraggio L., Barchetta S., Locorotondo G., Rebuzzi A.G., Crea F. (2010). Reversible coronary microvascular dys-function: A common pathogenetic mechanism in apical ballooning ot tako-tsubo syndrome. Eur. Heart J..

[29] Uchida Y., Egami H., Uchida Y., Sakurai T., Kanai M., Shirai S., Nakagawa O., Oshima T. (2010). Possible participation of endothelial cell apoptosis of coronary microvessels in the genesis of takotsubo cardiomyopathy. Clin. Cardiol..

[30] Suzuki H., Matsumoto Y., Kaneta T., Sugimura K., Takahashi J., Fukumoto Y., Takahashi S., Shimokawa H. (2014). Evidence for brain activation in patients with takotsubo cardiomyopathy. Circ. J..

[31] Klein C., Hiestand T., Ghadri J.-R., Templin C., Jäncke L., Hänggi J. (2017). Takotsubo syndrome—Predictable from brain imaging data. Sci. Rep..

[32] Templin C., Hänggi J., Klein C., Topka M.S., Hiestand T., Levinson R.A., Jurisic S., Lüscher T.F., Ghadri J.-R., Jäncke L. (2019). Altered limbic and autonomic processing supports brain-heart axis in Takotsubo syndrome. Eur. Heart J..

[33] Wang X., Pei J., Hu X. (2020). The Brain-Heart Connection in Takotsubo Syndrome: The Central Nervous System, Sympathetic Nervous System, and Catecholamine Overload. Cardiol. Res. Pract..

[34] Ghadri J.R., Wittstein I.S., Prasad A., Sharkey S., Dote K., Akashi Y.J., Cammann V.L., Crea F., Galiuto L., Desmet W. (2018). International Expert Consensus Document on Takotsubo Syndrome (Part I): Clinical characteristics, diagnostic criteria, and pathophysiology. Eur. Heart J..

[35] Stiermaier T., Walliser A., El-Battrawy I., Pätz T., Mezger M., Rawish E., Andrés M., Almendro-Delia M., Martinez-Sellés M., Uribarri A. (2022). Frequency, Characteristics, and Outcome of Takotsubo Syndrome Triggered by Positive Life Events. JACC Heart Fail..

[36] Fernández-Cordón C., Núñez-Gil I.J., de Miguel I.M., Pérez-Castellanos A., Vedia O., Almendro-Delia M., López-País J., Uribarri A., Duran-Cambra A., Martín-García A. (2023). Takotsubo Syndrome, Stressful Triggers, and Risk of Recurrence. Am. J. Cardiol..

[37] Yalçin F., Muderrisoğlu H. (2009). Tako-tsubo cardiomyopathy may be associated with cardiac geometric features as observed in hypertensive heart disease. Int. J. Cardiol..

[38] Yalçin F., Abraham M.R., Garcia M.J. (2024). Stress and Heart in Remodeling Process: Multiple Stressors at the Same Time Kill. J. Clin. Med..

[39] Spinelli L., Trimarco V., Di Marino S., Marino M., Iaccarino G., Trimarco B. (2009). L41Q polymorphism of the G protein coupled receptor kinase 5 is associated with left ventricular apical ballooning syndrome. Eur. J. Heart Fail..

[40] Limongelli G., Masarone D., Maddaloni V., Rubino M., Fratta F., Cirillo A., Ludovica S.B., Pacileo R., Fusco A., Coppola G.R. (2016). Genetics of Takotsubo Syndrome. Heart Fail. Clin..

[41] Previtali M., Repetto A., Camporotondo R., Citro R., Faggiano P., Bovelli D., Baldini E., Pasquetto G., Ascione L., Vignali L. (2011). Clinical characteristics and outcome of left ventricular ballooning syndrome in a European population. Am. J. Cardiol..

[42] Zilio F., Musella F., Ceriello L., Ciliberti G., Pavan D., Manes M.T., Selimi A., Scicchitano P., Iannopollo G., Albani S. (2024). Sex differences in patients presenting with acute coronary syndrome: A state-of-the-art review. Curr. Probl. Cardiol..

[43] Citro R., Rigo F., D’Andrea A., Ciampi Q., Parodi G., Provenza G., Piccolo R., Mirra M., Zito C., Giudice R. (2014). Echocardiographic correlates of acute heart failure, cardiogenic shock, and in hospital mortality in tako-tsubo cardiomyopathy. JACC Cardiovasc. Imaging.

[44] Citro R., Lyon A.R., Meimoun P., Omerovic E., Redfors B., Buck T., Lerakis S., Parodi G., Silverio A., Eitel I. (2015). Standard and advanced echocardiography in takotsubo (stress) cardiomyopathy: Clinical and prognostic implications. J. Am. Soc. Echocardiogr..

[45] Schneider B., Athanasiadis A., Schwab J., Pistner W., Gottwald U., Schoeller R., Toepel W., Winter K.-D., Stellbrink C., Müller-Honold T. (2014). Complications in the clinical course of tako-tsubo cardiomyopathy. Int. J. Cardiol..

[46] Tornvall P., Collste O., Ehrenborg E., Järnbert-Petterson H. (2016). A case-control study of risk markers and mortality in Takotsubo stress cardiomyopathy. J. Am. Coll. Cardiol..

[47] Ghadri J.R., Kato K., Cammann V.L., Gili S., Jurisic S., Di Vece D., Candreva A., Ding K.J., Micek J., Szawan K.A. (2018). Long-Term Prognosis of Patients With Takotsubo Syndrome. J. Am. Coll. Cardiol..

[48] Abe Y., Kondo M., Matsuoka R., Araki M., Dohyama K., Tanio H. (2003). Assessment of clinical features in transient left ventricular apical ballooning. J. Am. Coll. Cardiol..

[49] Prasad A., Lerman A., Rihal C.S. (2008). Apical ballooning syndrome (Tako-Tsubo or stress cardiomyopathy): A mimic of acute myocardial infarction. Am. Heart J..

[50] Ghadri J.R., Cammann V.L., Jurisic S., Seifert B., Napp L.C., Diekmann J., Bataiosu D.R., D’Ascenzo F., Ding K.J., Sarcon A. (2017). A novel clinical score (InterTAK Diagnostic Score) to differentiate takotsubo syndrome from acute coronary syndrome: Results from the International Takotsubo Registry. Eur. J. Heart Fail..

[51] Winchester D.E., Ragosta M., Taylor A.M. (2008). Concurrence of angiographic coronary artery disease in patients with apical ballooning syndrome (tako-tsubo cardiomyopathy). Catheter. Cardiovasc. Interv..

[52] Kurisu S., Inoue I., Kawagoe T., Ishihara M., Shimatani Y., Nakama Y., Maruhashi T., Kagawa E., Dai K., Matsushita J. (2009). Prevalence of incidental coronary artery disease in tako-tsubo cardiomyopathy. Coron. Artery Dis..

[53] Ghadri J.R., Wittstein I.S., Prasad A., Sharkey S., Dote K., Akashi Y.J., Cammann V.L., Crea F., Galiuto L., Desmet W. (2018). International Expert Consensus Document on Takotsubo Syndrome (Part II): Diagnostic workup, outcome, and management. Eur. Heart J..

[54] Santoro F., Mallardi A., Leopizzi A., Vitale E., Stiermaier T., Trambaiolo P., Di Biase M., Eitel I., Brunetti N.D. (2022). Stepwise approach for diagnosis and management of Takotsubo syndrome with cardiac imaging tools. Heart Fail. Rev..

[55] Nguyen T.H., Neil C.J., Sverdlov A.L., Mahadavan G., Chirkov Y.Y., Kucia A.M., Stansborough J., Beltrame J.F., Selvanayagam J.B., Zeitz C.J. (2011). N-terminal pro-brain natriuretic protein levels in Takotsubo cardiomyopathy. Am. J. Cardiol..

[56] Moady G., Yelin B., Sweid R., Atar S. (2023). C-Reactive Protein Can Predict Outcomes in Patients With Takotsubo Syndrome. Int. J. Heart Fail..

[57] Pelargonio G., La Rosa G., Di Stasio E., Narducci M.L., Rocco E., Angelini A., Pinnacchio G., Bencardino G., Perna F., Comerci G. (2021). Ventricular arrhythmias in Takotsubo Syndrome: Incidence, predictors and clinical outcomes. J. Cardiovasc. Med..

[58] Tse G., Gong M., Wong W.T., Georgopoulos S., Letsas K.P., Vassiliou V.S., Chan Y.S., Yan B.P., Wong S.H., Wu W.K. (2017). The T peak − T end interval as an electrocardiographic risk marker of arrhythmic and mortality outcomes: A systematic review and meta-analysis. Heart Rhythm..

[59] La Rosa G., Pelargonio G., Narducci M.L., Pinnacchio G., Bencardino G., Perna F., Follesa F., Galiuto L., Crea F. (2023). Prognostic value of the Tpeak-Tend interval for in-hospital subacute ventricular arrhythmias in tako-tsubo syndrome. Rev. Esp. Cardiol. (Engl. Ed.).

[60] Kurisu S., Inoue I., Kawagoe T., Ishihara M., Shimatani Y., Nakamura S., Yoshida M., Mitsuba N., Hata T., Sato H. (2004). Time course of electrocardiographic changes in patients with tako-tsubo syndrome: Comparison with acute myocardial infarction with minimal enzymatic release. Circ. J..

[61] Vizzardi E., Bonadei I., Piovanelli B., Bugatti S., D’Aloia A. (2014). Biventricular Tako-Tsubo cardiomyopathy: Usefulness of 2D speckle tracking strain echocardiography. J. Clin. Ultrasound.

[62] Stiermaier T., Reil J.-C., Sequeira V., Rawish E., Mezger M., Pätz T., Paitazoglou C., Schmidt T., Frerker C., Steendijk P. (2023). Hemodynamic Assessment in Takotsubo Syndrome. J. Am. Coll. Cardiol..

[63] Eitel I., von Knobelsdorff-Brenkenhoff F., Bernhardt P., Carbone I., Muellerleile K., Aldrovandi A., Francone M., Desch S., Gutberlet M., Strohm O. (2011). Clinical characteristics and cardiovascular magnetic resonance findings in stress (Takotsubo) cardiomyopathy. JAMA.

[64] Citro R., Pontone G., Pace L., Zito C., Silverio A., Bossone E., Piscione F. (2016). Contemporary Imaging in Takotsubo Syndrome. Heart Fail. Clin..

[65] Kohan A.A., Yeyati E.L., De Stefano L., Dragonetti L., Pietrani M., de Arenaza D.P., Belziti C., García-Mónaco R.D. (2014). Usefulness of MRI in takotsubo cardiomyopathy: A review of the literature. Cardiovasc. Diagn. Ther..

[66] Citro R., Okura H., Ghadri J.R., Izumi C., Meimoun P., Izumo M., Dawson D., Kaji S., Eitel I., Kagiyama N. (2020). Multimodality imaging in takotsubo syndrome: A joint consensus document of the European Association of Cardiovascular Imaging (EACVI) and the Japanese Society of Echocardiography (JSE). J. Echocardiogr..

[67] Juncà G., Teis A., Kasa G., Ferrer-Sistach E., Vallejo N., López-Ayerbe J., Cediel G., Bayés-Genís A., Delgado V. (2024). Timing of cardiac magnetic resonance and diagnostic yield in patients with myocardial infarction with nonobstructive coronary arteries. Rev. Espanola Cardiol. (Engl. Ed.).

[68] Liang K., Bisaccia G., Leo I., Williams M.G.L., Dastidar A., Strange J.W., Sammut E., Johnson T.W., Bucciarelli-Ducci C. (2023). CMR reclassifies the majority of patients with suspected MINOCA and non MINOCA. Eur. Heart J.—Cardiovasc. Imaging.

[69] Shanmuganathan M., Nikolaidou C., Burrage M.K., Borlotti A., Kotronias R., Scarsini R., Banerjee A., Terentes-Printzios D., Pitcher A., Gara E. (2024). Cardiovascular Magnetic Resonance Before Invasive Coronary Angiography in Suspected Non-ST-Segment Elevation Myocardial Infarction. JACC Cardiovasc. Imaging.

[70] Murakami T., Yoshikawa T., Maekawa Y., Ueda T., Isogai T., Sakata K., Nagao K., Yamamoto T., Takayama M. (2015). Gender Differences in Patients with Takotsubo Cardiomyopathy: Multi-Center Registry from Tokyo CCU Network. PLoS ONE.

[71] Budnik M., Nowak R., Fijałkowski M., Kochanowski J., Nargiełło E., Piątkowski R., Peller M., Kucharz J., Jaguszewski M., Gruchała M. (2020). Sex-dependent differences in clinical characteristics and in-hospital outcomes in patients with Takotsubo syndrome. Pol. Arch. Intern. Med..

[72] Natale E., Mistrulli R. (2023). Takotsubo syndrome: More frequent in women, more dangerous in men. Eur. Heart J. Suppl..

[73] Abusnina W., Elhouderi E., Walters R.W., Al-Abdouh A., Mostafa M.R., Liu J.L., Mazozy R., Mhanna M., Ben-Dor I., Dufani J. (2024). Sex Differences in the Clinical Outcomes of Patients With Takotsubo Stress Cardiomyopathy: A Meta-Analysis of Observational Studies. Am. J. Cardiol..

[74] Isogai T., Matsui H., Tanaka H., Fushimi K., Yasunaga H. (2016). Early beta-blocker use and in-hospital mortality in patients with takotsubo cardiomyopathy. Heart.

[75] Singh K., Carson K., Usmani Z., Sawhney G., Shah R., Horowitz J. (2014). Systematic review and meta-analysis of incidence and correlates of recurrence of takotsubo cardiomyopathy. Int. J. Cardiol..

[76] D’Ascenzo F., Gili S., Bertaina M., Iannaccone M., Cammann V.L., Di Vece D., Kato K., Saglietto A., Szawan K.A., Frangieh A.H. (2020). Impact of aspirin on takotsubo syndrome: A propensity score-based analysis of the InterTAK Registry. Eur. J. Heart Fail..

[77] Lyon A.R., Bossone E., Schneider B., Sechtem U., Citro R., Underwood S.R., Sheppard M.N., Figtree G.A., Parodi G., Akashi Y.J. (2016). Current state of knowledge on Takotsubo syndrome: A position statement from the Taskforce on Takotsubo Syndrome of the Heart Failure Association of the European Society of Cardiology. Eur. J. Heart Fail..

[78] de Gregorio C., Grimaldi P., Lentini C. (2008). Left ventricular thrombus formation and cardioembolic complications in patients with Takotsubo-like syndrome: A systematic review. Int. J. Cardiol..

[79] Mitsuma W., Kodama M., Ito M., Kimura S., Tanaka K., Hoyano M., Hirono S., Aizawa Y. (2010). Thromboembolism in Takotsubo cardiomyopathy. Int. J. Cardiol..

[80] Elkattawy O., Kunamneni S., Sutariya R., Ismail M., Mohamed O., Lee T.J., Javed J., Elkattawy S., Hossain A., Shamoon F. (2024). Pulmonary Embolism in Patients Admitted With Takotsubo Cardiomyopathy: Prevalence and Associated In-Hospital Adverse Events. Cureus.

[81] Baldetti L., Pagnesi M., Gallone G., Beneduce A., Belardinelli P., Melillo F., Spoladore R., Latib A., Colombo A., Giannini F. (2019). Thrombotic Complications and Cerebrovascular Events in Takotsubo Syndrome: A Systematic Review and Meta-analysis. Can. J. Cardiol..

[82] Santoro F., Stiermaier T., Tarantino N., De Gennaro L., Moeller C., Guastafierro F., Marchetti M.F., Montisci R., Carapelle E., Graf T. (2017). Left Ventricular Thrombi in Takotsubo Syndrome: Incidence, Predictors, and Management: Results From the GEIST (German Italian Stress Cardiomyopathy) Registry. J. Am. Heart Assoc..

[83] Madias J.E. (2016). If channel blocker ivabradine vs. beta-blockers for sinus tachycardia in patients with Takotsubo syndrome. Int. J. Cardiol..

[84] Sangen H., Imori Y., Tara S., Yamamoto T., Takano H., Shimizu W. (2018). Haemodynamic deterioration due to intra-aortic balloon counterpulsation in takotsubo cardiomyopathy. Eur. Heart J..

[85] Santoro F., Gil I.J.N., Stiermaier T., El-Battrawy I., Moeller C., Guerra F., Novo G., Arcari L., Musumeci B., Cacciotti L. (2023). Impact of intra-aortic balloon counterpulsation on all-cause mortality among patients with Takotsubo syndrome complicated by cardiogenic shock: Results from the German-Italian-Spanish (GEIST) registry. Eur. Heart J. Open.

[86] Redmond M., Knapp C., Salim M., Shanbhag S., Jaumdally R. (2013). Use of vasopressors in Takotsubo cardiomyopathy: A cautionary tale. Br. J. Anaesth..

[87] Antonini M., Stazi G.V., Cirasa M.T., Garotto G., Frustaci A. (2010). Efficacy of levosimendan in Takotsubo-related cardiogenic shock. Acta Anaesthesiol. Scand..

[88] Santoro F., Ieva R., Ferraretti A., Ienco V., Carpagnano G., Lodispoto M., Di Biase L., Di Biase M., Brunetti N.D. (2013). Safety and feasibility of levosimendan administration in takotsubo cardiomyopathy: A case series. Cardiovasc. Ther..

[89] Napp L.C., Westenfeld R., Møller J.E., Pappalardo F., Ibrahim K., Bonello L., Wilkins C., Pershad A., Mannino S.F., Schreiber T.L. (2022). Impella Mechanical Circulatory Support for Takotsubo SyndromeWith Shock: A Retrospective Multicenter Analysis. Cardiovasc. Revascularization Med..

[90] von Mackensen J.K.R., Zwaans V.I.T., El Shazly A., Van Praet K.M., Heck R., Starck C.T., Schoenrath F., Potapov E.V., Kempfert J., Jacobs S. (2024). Mechanical Circulatory Support Strategies in Takotsubo Syndrome with Cardiogenic Shock: A Systematic Review. J. Clin. Med..

[91] Santoro F., Ieva R., Musaico F., Ferraretti A., Triggiani G., Tarantino N., Di Biase M., Brunetti N.D. (2014). Lack of Efficacy of Drug Therapy in Preventing Takotsubo Cardiomyopathy Recurrence: A Meta-analysis. Clin. Cardiol..

[92] Stiermaier T., Rommel K.-P., Eitel C., Möller C., Graf T., Desch S., Thiele H., Eitel I. (2016). Management of arrhythmias in patients with Takotsubo cardiomyopathy: Is the implantation of permanent devices necessary?. Heart Rhythm..

[93] El-Battrawy I., Santoro F., Stiermaier T., Möller C., Guastafierro F., Novo G., Novo S., Santangelo A., Mariano E., Romeo F. (2020). Prevalence, management, and outcome of adverse rhythm disorders in takotsubo syndrome: Insights from the international multicenter GEIST registry. Heart Fail. Rev..

[94] Isogai T., Matsui H., Tanaka H., Makito K., Fushimi K., Yasunaga H. (2023). Incidence, management, and prognostic impact of arrhythmias in patients with Takotsubo syndrome: A nationwide retrospective cohort study. Eur. Heart J. Acute Cardiovasc. Care.

[95] Pelliccia F., Morgantini A., Rosati R. (2022). Takotsubo Syndrome: From Bench to Bedside and Bedside to Bench. J. Clin. Med..

